# The optimal window for reconstruction of the anterior cruciate ligament (ACL) with respect to quadriceps atrophies lies within 21 to 100 days

**DOI:** 10.1371/journal.pone.0296943

**Published:** 2024-02-01

**Authors:** Harald K. Widhalm, Alexander Draschl, Jannike Horns, Sebastian Rilk, Johannes Leitgeb, Stefan Hajdu, Patrick Sadoghi

**Affiliations:** 1 Department of Orthopedics and Traumatology, Clinical Division of Traumatology, Medical University of Vienna, Vienna, Austria; 2 Department of Orthopedics and Trauma, Medical University of Graz, Graz, Austria; 3 Division of Plastic, Aesthetic and Reconstructive Surgery, Department of Surgery, Medical University of Graz, Graz, Austria; 4 Department of Orthopaedic Surgery, Hospital for Special Surgery, NewYork-Presbyterian, Weill Medical College of Cornell University, New York, New York, United States of America; Uniklinik RWTH Aachen: Universitatsklinikum Aachen, GERMANY

## Abstract

**Purpose:**

The study’s objective was to determine the optimal window for anterior cruciate ligament (ACL) reconstruction with respect to quadriceps atrophies and clinical outcome.

**Methods:**

For this retrospective, comparative study, 115 patients aged under 35 were included, who received an ACL reconstruction between 2011 and 2016. They were divided into four groups, depending on the time to surgery, to determine the optimal window for reconstruction: (group 1: ≤21 d, group 2: <21d-56d≥, group 3: >56d-100d≥, group 4: >100d). Follow-up was performed one month postoperatively, after a mean of 4.9 (±5.3) months, and after a mean of 3.5 (±1.4) years. Primary endpoints included quadriceps muscle status, range of motion (ROM), pain, swelling, the International Knee Documentation Committee Subjective Knee Form (IKDC), the Lysholm-Score, the Knee Injury and Osteoarthritis Outcome Score (KOOS), and the Tegner-Activity-Scale (TAS).

**Results:**

Significantly more quadriceps atrophies were observed in group 1 and group 4, representing reconstructions earlier than 21 and later than 100 days (29% and 41% vs. 9%; p = 0.032). The measurements of knee extension (p = 0.082) and ROM (p = 0.123) were comparable in all groups. Group 1 showed the least pain (0% vs. 15%; p = 0.285) and swelling (0% vs. 23%; p = 0.077) compared to all other groups one month postoperatively. A comparison of postoperative clinical scores revealed no significant differences, with group 1 exhibiting the lowest TAS levels.

**Conclusion:**

In patients who underwent ACL reconstruction within three weeks or after more than 100 days, a significantly higher incidence of quadriceps atrophy was observed, possibly attributable to the initial inflammatory phase or the delayed reconstruction affecting quadriceps function. However, this impairment may not be observable in elite athletes who undergo reconstruction within hours of the injury.

## Introduction

Ruptures of the anterior cruciate ligament (ACL) are common injuries of the knee with an incidence of 1:3300 to 1:5000 [1–4]. An ACL reconstruction is supposed to restore the knee´s functional stability and ability to participate in sports [5,6]. In addition, it is supposed to prevent the knee from consecutive injuries caused by giving way symptoms [5,7–11].

The time interval from ACL injury to reconstruction and the time to surgery is debatable concerning the postoperative outcome [7,12–16]. However, despite many studies performed to identify the optimal timing of ACL reconstruction, there is no consensus in the literature yet [12,17]. Unfortunately, these studies vary in outcome measurements and surgical technics and have various definitions of early and delayed reconstruction [7,12–17].

One of the most interesting factors that might be affected by the time to surgery and affects the postoperative outcome is the condition of the thigh muscles, especially the quadriceps muscles of the ACL-reconstructed knee. Little is known about the influence of the time to surgery on these muscles, but previous studies revealed a correlation between quadriceps strength and patient satisfaction after ACL-reconstruction [18].

In addition, a superiority of early reconstructions in terms of speed of rehabilitation, the extent of movement, and patient-reported outcome measures (PROM) could be observed in previous investigations [12,19,20]. On the other hand, a potential correlation between early reconstructions and the occurrence of arthrofibrosis joint stiffness and persistent, terminal extension deficits was discussed [15,21–24].

Other outcome parameters of previous studies included knee pain, swelling [12,19,20], and the condition of the cartilage and menisci due to abnormal joint load of unstable knee joints [7,15,23,25–30]. The resulting recommendations regarding the optimal time-to-surgery differ strongly but agree on its influence on the outcome [8,15,20,23,29–31]. Furthermore, additional factors affecting the functional result after ACL reconstruction, such as the patient´s age, Body-Mass-Index (BMI), and sex, were identified [10,31,32].

To elucidate the optimal time-to-surgery, this study aimed to determine the optimal window for the reconstruction of the anterior cruciate ligament (ACL) with respect to quadriceps atrophies and clinical outcome.

## Materials and methods

This retrospective, monocentric study was performed upon approval of the Ethics Committee of the Medical University of Vienna (EK1117/2016). Written informed consent was obtained from all individuals included in the study. Parental/guardian consent was not obtained as minors were not included in the study. The patient data were retrieved from the hospital’s electronic health records (EHR) system, with the authors having access to information that could identify individual participants during the initial data collection. The data were accessed on May 12, 2018, for research purposes. To ensure the confidentiality and privacy of patients, each individual was assigned a unique ID, and the data was securely stored in an encrypted Excel file. This approach ensured the pseudonymization of patient data, separating it from any identifiable information after data collection.

**Fig 1:**
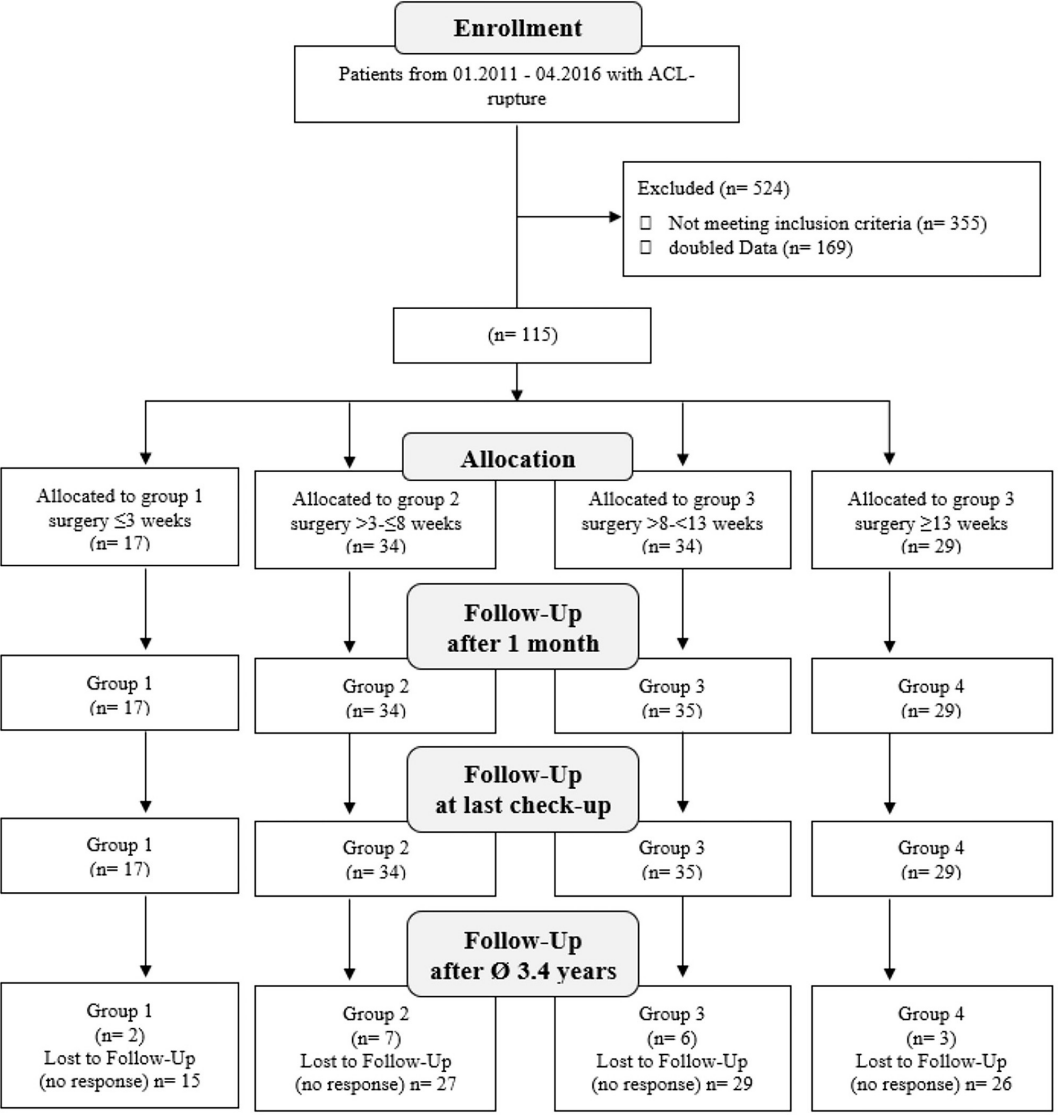
CONSORT Flow Diagram of included patients having undergone reconstruction of the anterior cruciate ligament (ACL) in group 1 (time from trauma until surgery < 21 days), group 2 (22 to 56 days), group 3 (57 to 100 days), and group 4 (after 100 days).

A total of 639 patients presented to our institute due to ruptures of their ACL between January 2011 and April 2016. After excluding patients who did not undergo ACL reconstruction, 115 remained and met the inclusion criteria for our study (Fig 1). Exclusion criteria were defined as combined injuries of the knee ligaments, both knees injured, age <18 years, age >35 years, chondral damage of Outerbridge level 3 and 4 [33], re-ruptures, and revision surgeries of the ACL.

Indication for the reconstruction of an ACL rupture was given based on the patient’s complaints, clinical symptoms, resulting limitations in everyday life, athletic requirement profile, and personal wishes according to previous investigations [34,35]. They were all treated with an autograft of the hamstring tendon in a single-bundle technique using the all-Inside technique, which has been standard at our division since 2011 [36]. All patients received standardized physical therapy and pain medication at follow-up according to a predefined protocol [37].

In our study, an impartial allocation coordinator, not involved in data collection or analysis, categorized the included patients into four groups to ensure that the investigators remained blinded to the assignment. To maintain homogeneity in terms of gender, BMI, graft choice, and age, patients were stratified based on these criteria. In the first group, patients were treated within 21 days, patients of group 2 from 22 to 56 days, those of group 3 from 57 until 100 days, and those of group 4 after more than 100 days. The definition of 3 weeks as an early reconstruction was common in previous studies [16,20,22,38,39]. The other groups were chosen regarding the quartiles of the patient count for having a comparable number of patients suitable for statistical analysis. From the data of the included patients, the time-to-surgery, sex, age, BMI, leg side, graft choice, and outcome parameters were listed. From the data of the patients, who were not suitable for inclusion in the study, the respective reasons for exclusion were collected and summed up.

### Outcome evaluations

For quantifying the postoperative outcome, it was subclassified into an objective and a subjective part. The objective outcome measures comprised several parameters, including the assessment of quadriceps muscle atrophy through two thigh circumference measurements of the affected and contralateral leg conducted preoperatively and at the final follow-up, both taken 10 cm proximal to the patellar base. Moreover, postoperative assessments were conducted twice for Range-of-Motion (ROM), extension deficits, knee pain, swelling, and effusion: first, one month after surgery, and then again during the final follow-up. The ROM was measured by independent investigators using a goniometer, with the patient in a supine position. Since the retrospective data rarely provided information concerning the ROMs of the contralateral knees, absolute degree values of the injured knees had to be used. A ROM of >120° was considered an excellent result, 90 to 120° an average result, and <90° was defined as a poor result. The extension values were also analyzed individually, as some studies described an association between early reconstructions and postoperative extension deficits [15,22–24].

Since the objective result measurements of ROM and stability do not necessarily correspond to the subjective functionality of a knee joint after cruciate ligament reconstruction [40], the subjective outcome was evaluated by using four standardized questionnaires frequently used in studies: the International Knee Documentation Committee Subjective Knee Form (IKDC) [19,40,41], the Lysholm-Score [23,42,43], the Knee Injury and Osteoarthritis Outcome Score (KOOS) [12,44,45], and the Tegner-Activity-Scale (TAS) [42,46]. We opted to implement these subjective outcomes in alignment with a previous review’s suggestion, aiming to ensure the validity and reliability of the outcome measurements applied in this study for patients undergoing ACL reconstruction [47]. Additionally, the patients’ estimation of the ROM of the ACL-reconstructed knee was used for analysis.

The IKDC, Lysholm-Score, and KOOS inquired about knee function in everyday activities and specific situations. In each questionnaire, it was possible to score up to 100 points when having a perfectly functional knee. With decreasing score points, the functional limitations and symptoms of the injured knee joint increase. The TAS, as a complementary measure to the Lysholm-Score, was used to grade sports activities from 0 (sick leave or disability) to 10 (competitive sports level) [42].

## Statistical analysis

All data were analyzed regarding the time-to-surgery groups. The software SPSS Version 26 and Excel were used for the statistical analysis. The distribution of ordinally scaled parameters as well as categorical parameters, were given with absolute frequencies and as percentages. For the metrical parameters, the count of valid participants (n), the arithmetic mean, standard deviation, and median values were listed in a table. This was conducted for the participants as a whole and for each group itself. For all statistical tests, the p-values were listed in a table. Statistical significance was set at a p-value of <0.05, and the statistical power was 80%.

The four groups were analyzed with Chi^2^-Test regarding the following nominal parameters of the objective outcome: quadriceps atrophy, extension deficits, Range-of-Motion, knee pain and -swelling, and joint effusion. If the expected count for each space of the statistical table was <5, the Likelihood-Quotient results were considered more reliable than those of the Chi^2^-Test.

To examine a correlation of the subjective outcomes, the metrical score points of the questionnaires were correlated with the ordinal scaled groups using the Kruskal-Wallis-Test. Post hoc power was calculated according to the magnitude of the difference of the primary endpoint quadriceps atrophy with a 5% difference set as clinically significant, according to Hoenig and Heisey [48].

## Results

The patients`demographics are detailed in Table 1. The group distribution was 17 patients in group 1, 34 in group 2, 35 in group 3, and 29 in group 4.

**Table 1:** Patient characteristics^a^.

Outcome parameters		Group 1 (n = 17)		Group 2 (n = 34)		Group 3 (n = 35)		Group 4 (n = 29)		Missing, n (%)	
TS (n = 115)										0	(0%)
	Mean (±SD) [days]	10.5	(±5.4)	41.8	(±10.4)	76.4	(±12.5)	225.8	(±140.3)		
Gender (n = 115)										0	(0%)
	Male, n (%)	9	(53%)	25	(73%)	28	(80%)	23	(79%)		
	Female, n (%)	8	(47%)	9	(27%)	7	(20%)	6	(21%)		
Age (n = 115)										0	(0%)
	≤25 years, n (%)	9	(53%)	18	(53%)	18	(51%)	15	(52%)		
	>25≤35 years, n (%)	8	(47%)	16	(47%)	17	(49%)	14	(48%)		
BMI (n = 85)										30	(26%)
	<20, n (%)	1	(8%)	2	(8%)	1	(4%)	4	(19%)		
	20–25, n (%)	8	(62%)	15	(63%)	17	(63%)	14	(67%)		
	>25, n (%)	4	(30%)	7	(29%)	9	(33%)	3	(14%)		
Affected leg (n = 115)										0	(0%)
	right, n (%)	4	(23%)	15	(40%)	12	(34%)	15	(52%)		
	left, n (%)	13	(77%)	19	(60%)	23	(66%)	14	(48%)		
Graft choice (n = 115)										0	(0%)
	S	8	(47%)	17	(50%)	14	(40%)	13	(45%)		
	SG	9	(53%)	17	(50%)	21	(60%)	16	(55%)		

^a^ Mean values (±SD) or absolute and relative frequencies for each group unless otherwise indicated.

TS = Time to surgery.

SD = Standard deviation.

S, SG = semitendinosus tendon autograft, semitendinosus and gracilis tendon autograft.

**Fig 2:**
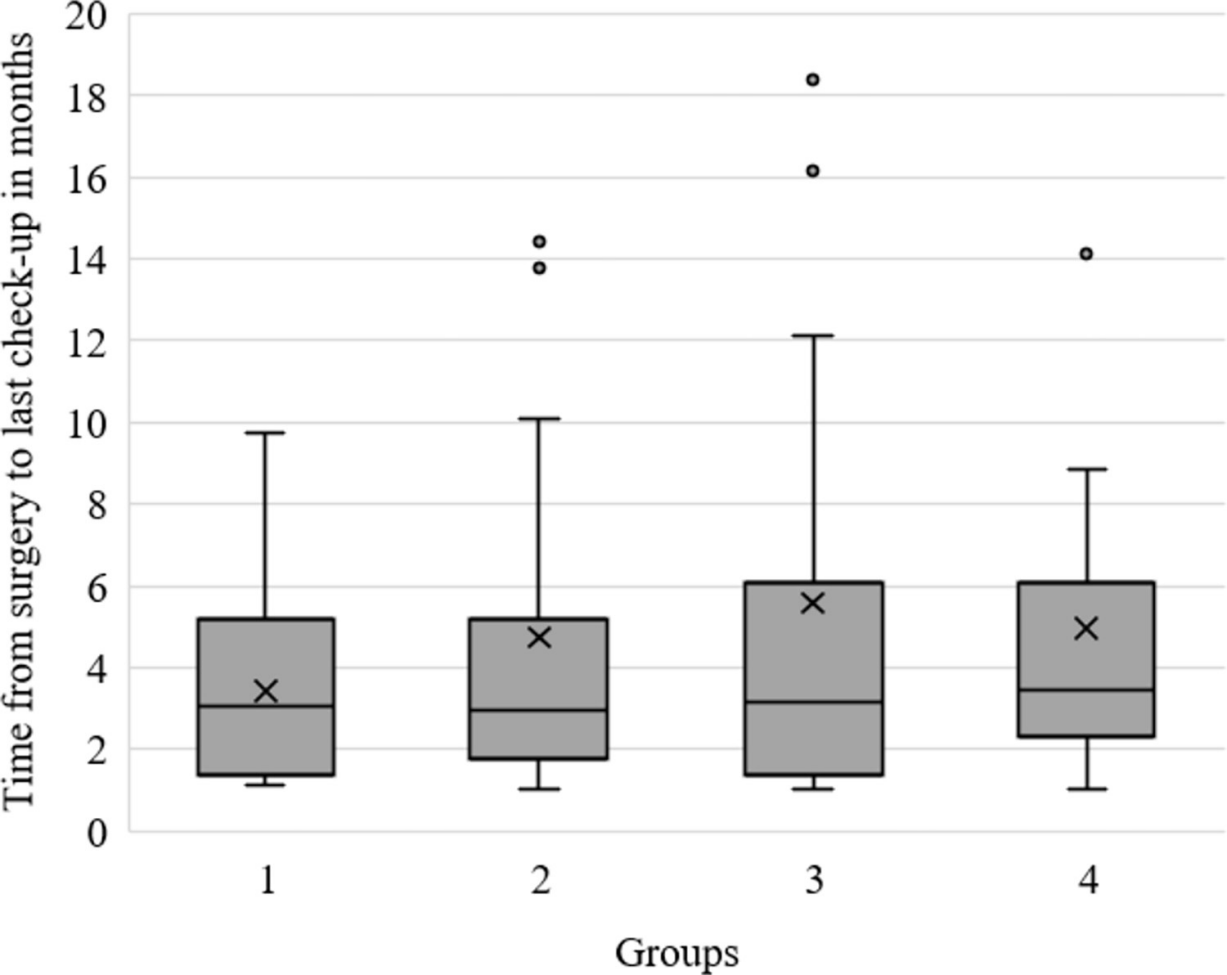
Boxplots showing time from ACL reconstruction to last check up for each group.

The time interval from surgery to the last check-up varied from 1–30 months, with a mean of 4.8 months and a median of 3 months (Fig 2).

Table 2 details the comparisons between the groups and the outcome parameters. On average, 22% of all patients who underwent surgery exhibited a decrease in thigh muscle circumference compared to the contralateral leg at the final measurement point.

**Table 2:** Outcome parameters ^a^.

Outcome parameters		Patient count		Group 1		Group 2		Group 3		Group 4		*p* value
Quadriceps atrophy												
	Last date	25/112	(22%)	5/17	(29%)	3/33	(9%)	5/33	(15%)	12/29	(41%)	0.013
	Survey	9/15	(60%)	1/1	(100%)	2/5	(40%)	4/5	(80%)	2/2	(100%)	<0.001
Extension deficits												
	1 month postoperatively	32/115	(28%)	6/17	(35%)	13/34	(38%)	10/35	(29%)	3/29	(10%)	0.075
	Final follow-up	14/115	(12%)	1/17	(6%)	6/34	(18%)	6/35	(17%)	1/29	(3%)	0.082
ROM												
	<90° 1 month postoperatively	16/115	(14%)	3/17	(18%)	9/34	(26%)	1/35	(3%)	3/29	(10%)	0.089
	90–120° 1 month postoperatively	77/115	(67%)	12/17	(71%)	20/34	(59%)	27/35	(77%)	18/29	(62%)	0.089
	>120° 1 month postoperatively	22/115	(19%)	2/17	(11%)	5/34	(15%)	7/35	(20%)	8/29	(28%)	0.089
	<90° at final follow-up	6/115	(5%)	0/17	(0%)	2/34	(6%)	3/35	(9%)	1/29	(3%)	0.123
	90–120° at final follow-up	39/115	(34%)	9/17	(47%)	7/34	(21%)	15/35	(43%)	8/29	(28%)	0.123
	>120° at final follow-up	70/115	(61%)	8/17	(53%)	25/34	(73%)	17/35	(48%)	20/29	(69%)	0.123
Knee pain												
	1 month postoperatively	9/98	(9%)	0/15	(0%)	4/27	(15%)	3/32	(9%)	2/24	(8%)	0.285
	Final follow-up	22/112	(24%)	3/17	(18%)	8/33	(25%)	6/33	(18%)	5/29	(17%)	0.892
Knee joint effusion												
	1 month postoperatively	31/97	(40%)	5/15	(33%)	9/26	(35%)	10/32	(31%)	7/24	(29%)	0.979
	Final follow-up	9/112	(8%)	2/17	(12%)	3/33	(9%%)	2/33	(6%)	2/29	(7%)	0.903
Swelling												
	1 month postoperatively	13/97	(32%)	0/15	(0%)	6/26	(23%)	5/32	(16%)	2/24	(8%)	0.077
	Final follow-up	9/112	(8%)	0/17	(0%)	4/33	(12%)	4/33	(12%)	1/29	(3%)	0.159

^*a*^ *Mean values for each group and relative frequencies based on total patient counts*.

**Fig 3:**
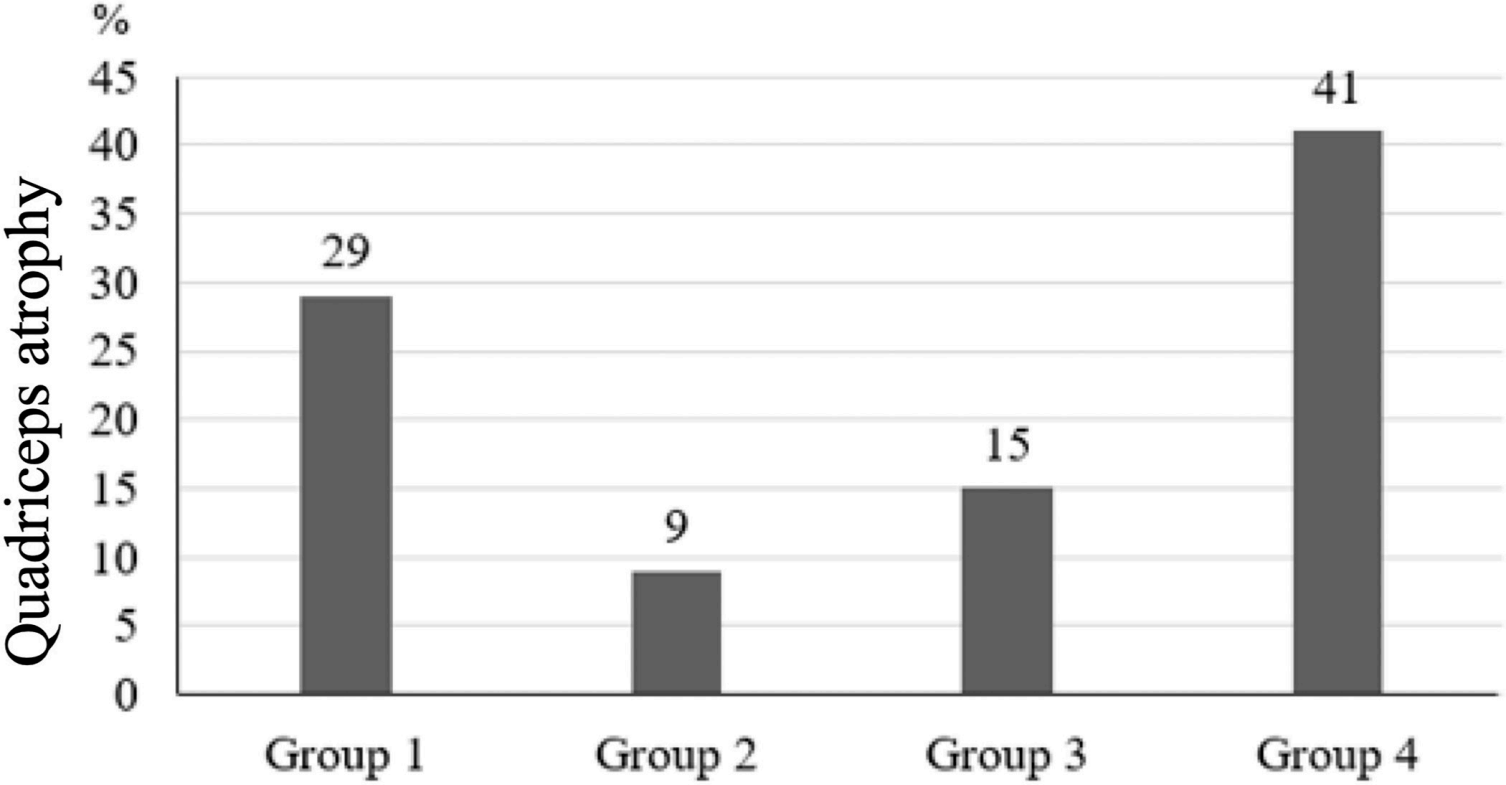
Quadriceps atrophy in percent (%) for each group.

Quadriceps atrophy was seen most within groups 1 and 4 (Fig 3). This result was statistically significant and clinically relevant (greater than 5%). In the online questionnaire, 60% of patients noted a smaller thigh circumference in the ACL-reconstructed leg.

**Fig 4:**
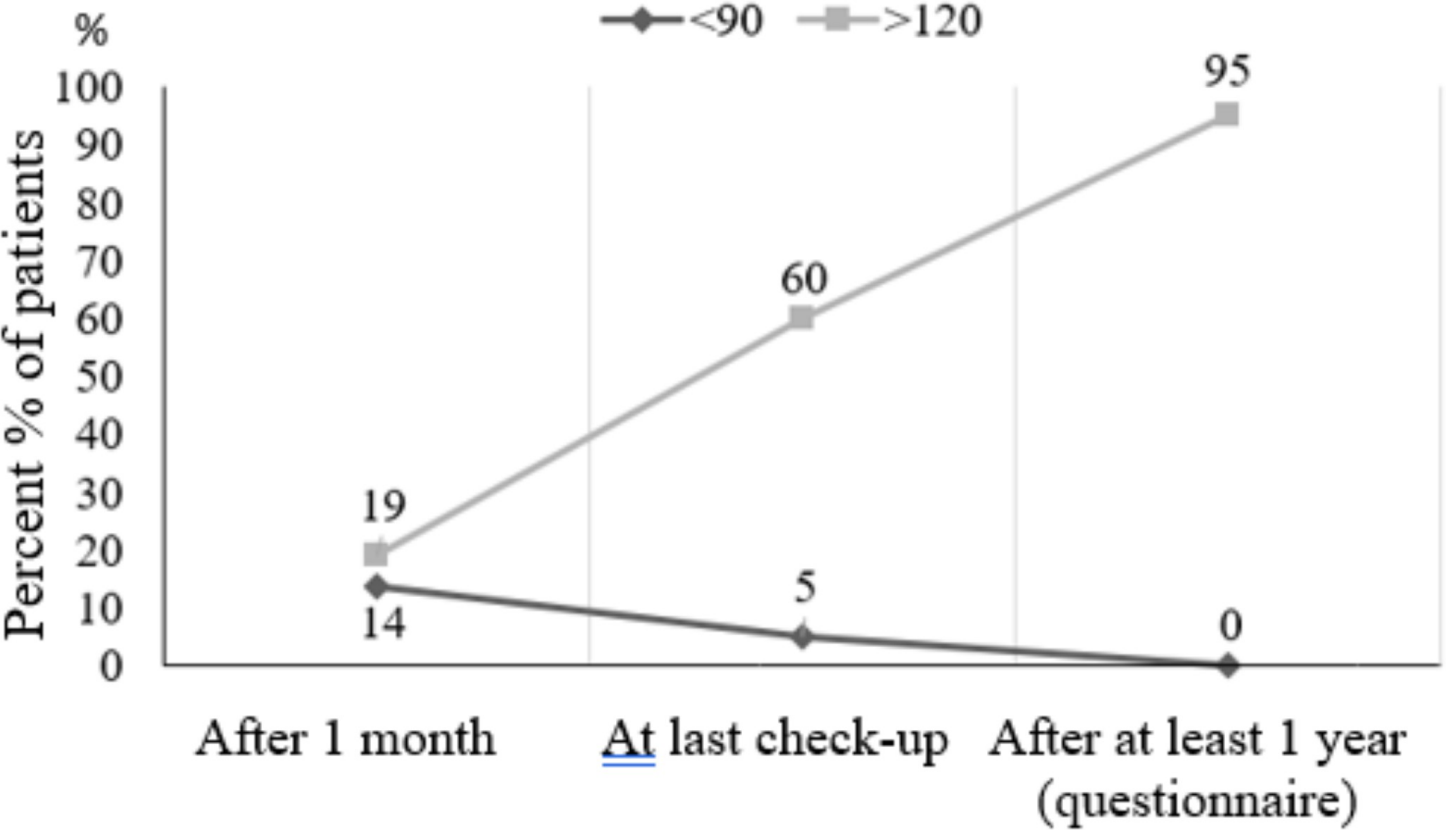
Range-of-Motion at the three follow-ups.

In total, the Range-of-Motion (ROM) stretch deficits improved from one month to the last measured date. The range of motion continued improving until the survey was conducted (Fig 4). The self-assessed general mobility of the knee joint was >120° in 95% of the cases. There was no statistically significant difference after one month and later.

In percentage terms, group 1 showed the lowest knee pain and swelling rate after one month compared to the other groups. These differences were not statistically significant.

**Fig 5:**
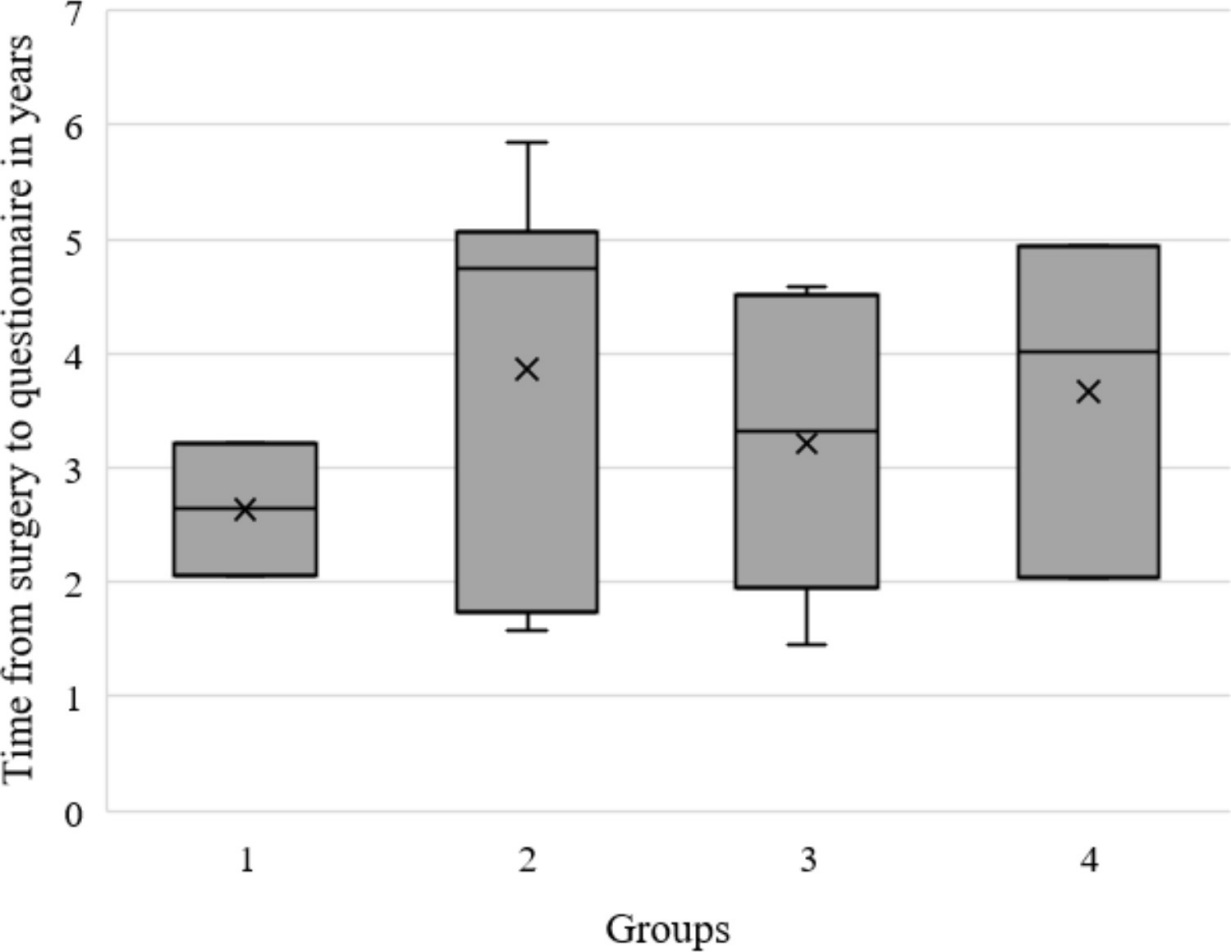
Boxplots showing time from ACL-reconstruction to participating in the survey for each group.

The time interval from surgery to completing the questionnaire was at least one year, with a mean of 3.4 years (Fig 5).

In the three questionnaires, except for the mean TAS, patients of group 4 achieved the highest average scores (Table 3). There were no statistically significant differences in the results of the questionnaires between the groups. Group 1 achieved the lowest TAS levels postoperatively.

**Table 3:** Questionnaires^a^.

Outcome parameters	Group 1 (n = 2)	Group 2 (n = 8)	Group 3 (n = 4)	Group 4 (n = 9)	*p* value
**IKDC**	84.5 (±20.3)	86.7 (±9.3)	84.7 (±13.8)	90.1 (±6.4)	0.398
**Lysholm-Score**	88.1 (±7.7)	88.1 (±6.3)	90.1 (±4.9)	90.8 (±3.8)	0.659
**KOOS**	85.5 (±6.4)	82.0 (±14.4)	88.0 (±10.1)	90.3 (±5.6)	0.974
**TAS-Level**	3.0 (±2.8)	5.4 (±1.5)	7.2 (±0.4)	4.3 (±2.5)	>0.050

^a^ Mean values (±SD) for each group.

Physical therapy was provided for an average of 3.2 months, and unsupervised home exercises were practiced for an average of 6.2 months [37]. A mean of 10.3 months was needed to reach the existing knee condition (full ROM and clinical stability) when completing the questionnaire.

Post hoc power analysis, according to Hoenig and Heisey, revealed that according to the magnitude of differences in quadriceps atrophies between groups, a power greater than 80% could be reached, indicating sufficient power. In addition, both interclass as well as intraclass correlation analyses revealed excellent agreement with values greater than 0.8.

## Discussion

The most important findings of the present study were that significant differences in the outcome between early or delayed reconstruction could be proven. Statistically significant and clinically relevant differences were observed regarding quadriceps atrophies and noteworthy differences in the results of other outcome parameters.

Several studies reported that an ACL injury leads to differences in the strength and volume of the thigh muscles compared to the contralateral knee [26,49–52]. Possibly due to the loss of mechanoreceptor feedback from the ACL to the thigh muscles, these get less often activated and lose volume and strength [53,54]. To prevent a loss of muscle volume, intense pre- and postoperative physiotherapy focusing on the quadriceps muscle strength and proprioception is, therefore, indispensable [49,55]. As stated by Shelbourne et al. [52] the time-to-surgery had an influence on the rate of quadriceps atrophies with the least atrophies in early reconstructions. Their findings are supported by a study conducted by Wenning et al. who demonstrated an increase in loss of thigh muscle strength in delayed ACL reconstruction cases, emphasizing to seek for an early ACL reconstruction within the first 12 weeks after the injury [24].Opposite to these findings [52] in the present study, the patients of group 1 and group 4 showed the highest rate of quadriceps atrophies. This finding might be due to the fact that we did not further differentiate between reconstructions within the first hours after the injury in group 1, usually performed in elite athletes, but included all patients in the period until the first 21 days after injury. Immediate reconstruction for elite athletes could have been performed prior to the initiation of the inflammatory process and, therefore, not lead to quadriceps atrophy, as stated by Shelbourne et al. [52]. However, in our presented data, we included patients having undergone the procedure in the second and third week after the initial trauma, who were in the strong inflammatory phase. The higher rate of atrophy in group 4, having undergone the reconstruction over 100 days after trauma, is evident due to loss of muscle mass due to loss of function, which was not that evident within 21 and 100 days after the initial trauma in our study group.

The last date of check-up was in the median after 3 months, and patients stated that it took in the mean 10 months to reach the final stage of rehabilitation of their ACL-reconstructed knees. Unfortunately, no data regarding the time interval of the preoperative physiotherapy or duration of conservative rehabilitation therapy were listed, so that reasons for the high rate of quadriceps atrophies in group 4 can only be assumed, as mentioned before. The patients received their ACL reconstruction after a mean time of half a year with a maximum of one and a half years. Maybe their preoperative physiotherapy wasn’t concepted or conducted for such a long time, or they started a conservative therapy trial that failed to restore knee stability and thigh strength.

Interestingly, despite the high rates of quadriceps atrophies in groups 1 and 4, they showed at both measured times the least pain and swellings and reached the highest activity levels postoperatively. This leads to the assumption as Arangio et al. [26] noted that the measurements of the quadriceps circumference don’t correlate with its strength and function after ACL reconstruction. Nawasreh et al. [56] stated that young patients with ACL rupture and early reconstruction returned to sport more quickly and were more likely to achieve a similar level of sport as before the trauma, both in the publication of Bottoni et al. [20] and in our study, there was a tendency that early reconstruction could allow higher activity levels postoperatively. Nevertheless, in line with previous studies [20,57,58], no statistically significant difference between all groups was found.

Bottoni et al. [20] additionally found a superiority of early reconstructions within 17 days regarding the speed of rehabilitation and extent of movement. On the contrary, there is evidence that an early ACL reconstruction can lead to arthrofibrosis [15,59]. Conversely, Agarwal et al. [60] found that postponing ACL reconstruction by a minimum of 6 weeks in patients below 40 years of age is linked to a 65% decrease in the likelihood of requiring surgical intervention for arthrofibrosis. Moreover, delaying the procedure by at least 10 weeks in patients aged 40 years and above is associated with a 35% reduction in the risk of surgical intervention for arthrofibrosis [60]. In line with other studies [20,21,38], we noted no differences between the groups regarding extension deficits and Range-of-Motion after one month and at the last measurement date. The ROM improved with time, and when the questionnaire was conducted, almost all patients estimated their knees’ movement as over 120°.

In the survey of the subjective outcome, most patients reported that their ACL-reconstructed knee joint would not represent any impairment in everyday life. In the standardized questionnaires, the results of all groups were comparable with the best scores in group 4. This corresponded to the results of Ranjan et al. [61] and Petersen et al. [23], where the patients who received a delayed ACL reconstruction also showed better results in the IKDC and Lysholm-Score. As in the present study, other investigations didn’t reveal any statistically significant differences in the score values of the questionnaires IKDC, Lysholm-Score, and KOOS [57,62,63].

This study’s strengths were the different follow-up times, a homogenous patient collective regarding BMI, age, and graft choice owing to stratified sampling, the many different outcome parameters collected, and the combination of subjective and objective variables. However, there were some limiting factors in the presented study. First, it was a retrospective study, suffering from the possible influence of selection bias, as there were strict exclusion criteria such as age <18 and age >35 years, severe cartilage damage, combined ligament injuries, etc. Next, the choice of time-to-surgery was not made according to a standardized procedure but rather on a patient-specific basis and after consultation with different surgeons with possibly different experiences, who also influenced the surgical method, graft selection, surgical procedure, postoperative procedure, and thus the reconstruction result. Furthermore, we want to point out that there is a difference in patient count regarding quadriceps atrophy and functional outcome assessments at the final follow-up, which is evident due to the fact that 17 patients did not complete both, the final evaluation and the survey. In addition, the retrospective outcome evaluations are dependent on the examiner. However, we took measures to blind the examiner conducting measurements, and an independent examiner was employed to assess the measurements independently to minimize potential bias and enhance the reliability of our study results. Finally, when the patients assessed their ROM in the questionnaire, there was a risk for them to either significantly overestimate or underestimate their ROM based on their subjective judgment.

## Conclusions

The occurrence of quadriceps atrophy was significantly higher in patients with anterior cruciate ligament (ACL) reconstruction within 3 weeks or after more than 100 days, possibly due to the initial inflammatory phase or delayed reconstruction impairing quadriceps function. Further research into the specific mechanisms underlying quadriceps atrophy in patients with ACL reconstruction should be emphasized. Studying postoperative inflammation’s effects on early muscle function and assessing the long-term impact of delayed reconstruction could optimize rehabilitation protocols, and prospective studies with larger samples and extended follow-up may further clarify factors influencing quadriceps atrophy in ACL reconstruction patients.

## Abbreviations

ACL: Anterior cruciate ligament
BMI: Body-Mass-Index
IKDC: International Knee Documentation Committee Subjective Knee Form
KOOS: Knee Injury and Osteoarthritis Outcome Score
MRI: Magnetic Resonance Imaging
PROM: Patient-reported outcome measures
ROM: Range of motion
TAS: Tegner-Activity-Scale
